# Assessing the Association of Serum Zinc and Copper Levels With Disease Activity Using the Juvenile Arthritis Disease Activity Score (JADAS) in Juvenile Idiopathic Arthritis

**DOI:** 10.7759/cureus.93195

**Published:** 2025-09-25

**Authors:** Tanzida Sultana, Sahida Sultana, Muhammed Waliur Rahman, Mujammel Haque, Mohammad Imnul Islam

**Affiliations:** 1 Pediatrics, Bangladesh Medical University, Dhaka, BGD; 2 Biochemistry and Molecular Biology, Bangladesh Medical University, Dhaka, BGD

**Keywords:** copper, disease activity, jadas, juvenile arthritis disease activity score, juvenile idiopathic arthritis, zinc

## Abstract

Background

Alteration of zinc (Zn) and copper (Cu) levels could serve as a useful biomarker for assessing disease activity status in juvenile idiopathic arthritis (JIA) patients.

Objectives

To assess the serum zinc and copper levels and their association with disease activity status using the Juvenile Arthritis Disease Activity Score (JADAS) in JIA patients.

Methods

This was a prospective observational study. JIA patients who fulfilled the International League of Associations for Rheumatology (ILAR) classification criteria were enrolled in this study. Detailed history, clinical examination, and laboratory investigations were recorded. Serum zinc and copper levels were assessed at the initial visit, the 12th week, and the 24th week of follow-up. Disease activity status was assessed using the JADAS. Statistical analysis was used with appropriate tests (paired t-test, ANOVA) in SPSS 27.0 (IBM Corp., Armonk, NY).

Results

At enrollment, all JIA patients were in high disease activity, according to the JADAS score. Among them, seven (15.6%) and 23 (51.1%) of JIA patients showed inactive disease states at the 12th week and 24th week of follow-up. Serum zinc level was initially lower than normal and then gradually increased at 12 weeks and 24 weeks, respectively. However, these changes were not statistically significant. Conversely, mean serum copper levels were high at the initial visit, which significantly decreased by the 12th week (p < 0.05) and 24th week (p < 0.05). The analysis of serum zinc and copper levels revealed that while zinc showed no significant variation across disease activity states. JIA patients with high disease activity had notably higher mean serum copper levels (p < 0.05) compared to those with moderate, low, and inactive disease.

Conclusion

Serum zinc and copper levels vary with JIA disease activity. In active disease states, serum Cu levels were significantly increased, while serum Zn levels were decreased; however, the difference was not statistically significant.

## Introduction

Juvenile idiopathic arthritis (JIA) is the most common rheumatic illness of childhood, characterized by chronic inflammation of one or more joints in children and adolescents, resulting in short and long-term morbidity and disability [1]. According to the International League of Associations for Rheumatology (ILAR), JIA is defined as definite arthritis of unknown etiology that begins before the 16th year of age and persists for at least six weeks [2].

The Juvenile Arthritis Disease Activity Score (JADAS-27) is the most widely accepted disease scoring system for patients with JIA, identifying levels of disease activity [3]. JADAS-27 is calculated by the sum of four components: physician’s global assessment of disease activity by visual analog scale (VAS); parents/patients' global assessment of well-being by VAS, counts of joints with active disease, and erythrocyte sedimentation rate (ESR) [3].

Trace elements such as zinc (Zn) and copper (Cu) are crucial catalytic cofactors for several enzymes, structural proteins, and transcription factors. In addition, they are essential elements for the immune system and for the integrity of the articular tissues [4,5].

In addition, Zn constitutes a structural element of alkaline phosphatase and stimulates its synthesis in osteoblasts, playing an important role in bone mineralization, and it is responsible for the latency of metalloproteinases [6].

As a part of chronic inflammatory response, the induction of interleukin-1 synthesis increases the metallothionein-mediated hepatic uptake of serum Zn, attributed to hypozincemia in JIA patients [7].

Copper and zinc can activate many key enzymes in cellular metabolism. They are constituents of the superoxide-dismutase enzyme, which performs intracellular antioxidant functions [8].

Copper is a constituent of ceruloplasmin, a powerful extracellular antioxidant enzyme [9]. In chronic inflammatory conditions like JIA, the production of interleukin-1 increases, which causes upregulation of the ceruloplasmin (acute phase reactant) gene and synthesis in the liver, and subsequently the level of ceruloplasmin-Cu complexes in the blood [10].

The anti-inflammatory effects of copper and zinc have been documented in different studies [11]. There are a few studies that were conducted to examine the role of zinc and copper levels in JIA patients. An Egyptian study found alteration of copper and zinc levels in JIA patients relative to controls [12]. Variation of serum copper and zinc is probably a defense response against JIA, and increased copper may be due to inflammation. So, these trace elements could serve as biomarkers for disease activity [13].

No such study was conducted in our country related to serum Zn and Cu and their association with disease activity. This study aimed to assess serum Zn and Cu levels and their association with disease activity in JIA patients using the JADAS-27.

## Materials and methods

This prospective observational study was carried out in the Pediatric Rheumatology Division, Department of Pediatrics, Bangladesh Medical University (BMU), from August 2022 to July 2023. A total of 45 children under 16 years of age, who met the ILAR criteria for JIA during the study period, were included in this study. JIA patients having acute infection, chronic liver disease, renal failure, or malignancy, and who had taken zinc supplements within the past month, were excluded from the study. History, clinical examination, and relevant investigations were recorded in the pre-designated questionnaire.

After taking informed written consent, blood samples were collected for complete blood count, erythrocyte sedimentation rate (ESR), serum creatinine, serum alanine aminotransferase (ALT), serum zinc, serum copper, and urine analysis. The serum Zn and Cu analyses were done at the Department of Biochemistry, Bangabandhu Sheikh Mujib Medical University (BSMMU), by the spectrophotometric method with INDIKO PLUS Drug Analyzer (Thermo Fisher Scientific, Waltham, MA). All participants were assessed for disease activity using the JADAS-27, and investigations were sent to estimate serum zinc and serum copper levels at the initial visit and at the subsequent 12th and 24th weeks of follow-up.

This study was conducted with prior approval from the Institutional Review Board, Bangabandhu Sheikh Mujib Medical University, Dhaka, Bangladesh (BSMMU/2022/6735). The entered data were checked, verified, and analyzed using SPSS version 27 (IBM Corp., Armonk, NY). Appropriate statistical tests (e.g., paired t-test and ANOVA test) were applied for data analysis. Qualitative data were expressed as frequency and percentage. A p-value less than 0.05 was considered significant.

## Results

The mean age of the study population was 9.8 ± 3.3 years. More than half of the study population (23, 51.1%) was within the 10-16 years of age group. In this cohort, 24 (53.3%) were males, and 21 (46.7%) were females. The mean age of disease onset was 7.57 ± 3.80 years (Table 1).

**Table 1:** Baseline characteristics of the study population (n = 45).

Variables	Number (n)	Percentage (%)
Age group		
4 years	4	8.9
5-9 years	18	40.0
10-16 years	23	51.1
Mean ± SD (years)	9.8±3.32	
Gender		
Male	24	53.3
Female	21	46.7
Age of disease onset (years), mean ± SD	7.57±3.80	

**Figure 1:**
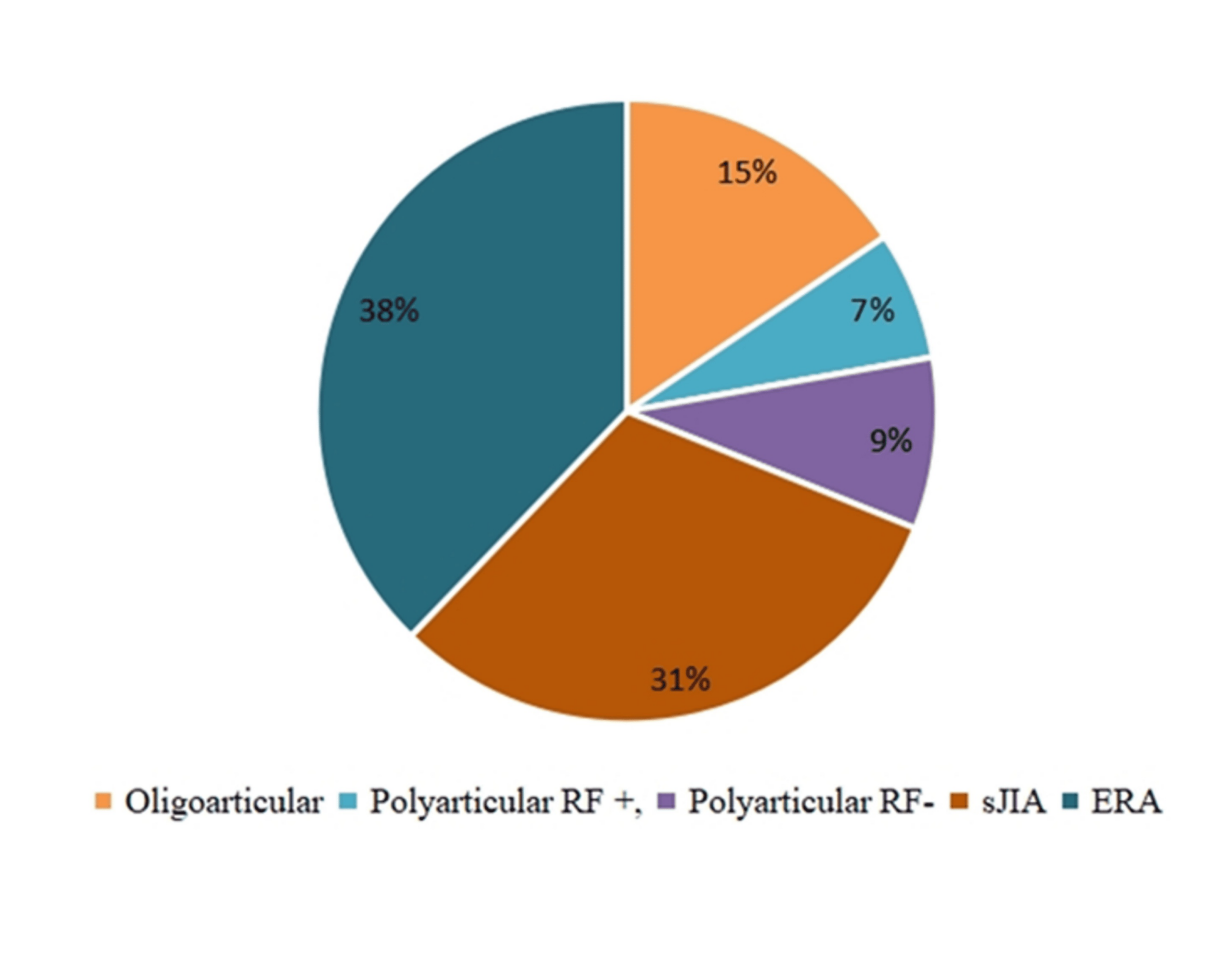
Pie chart showing types of JIA among the study population. JIA: juvenile idiopathic arthritis; RF: rheumatoid factor; sJIA: systemic juvenile idiopathic arthritis; ERA: enthesitis-related arthritis.

Among the different subtypes of JIA, enthesitis-related arthritis (ERA) was the most prevalent (17, 38%), followed by systemic juvenile idiopathic arthritis (sJIA) (14, 31%). Oligoarticular JIA represented seven (15%), while polyarticular rheumatoid factor (RF)-positive and polyarticular RF-negative JIA were seen in three (7%) and four (9%) patients, respectively (Figure 1).

The disease activity status of JIA cases showed that all cases were classified as high disease activity at the initial visit. Gradually, with time, 23 (51.1%) of the patients became inactive, while only three (6.7%) remained in the high disease activity category (Table 2).

**Table 2:** Disease activity status of juvenile idiopathic arthritis cases at initial visit and subsequent 12th and 24th week follow-up (n = 45).

Disease activity status	0 week, n (%)	12^th^ week, n (%)	24^th^ week, n (%)
				Inactive disease	0 (0.0)	7 (15.6)	23 (51.1)
Low disease activity	0 (0.0)	13 (28.9)	12 (26.7)
Moderate disease activity	0 (0.0)	11 (24.4)	7 (15.6)
High disease activity	45 (100.0)	14 (31.1)	3 (6.7)

The comparison of mean serum zinc and copper levels with time among the study population is shown in Table 3. Serum Zn levels increased gradually over time, but the change was not statistically significant. In contrast, serum copper levels showed a significant decrease over time at the 12th week (p < 0.05) and 24th week (p < 0.05), which was statistically significant.

**Table 3:** Comparison of mean serum zinc and copper levels at baseline, 12th week, and 24th week among the study population (n = 45).

Parameters	0 week	12^th^ week	24^th^ week	P-value by paired t-test	
				0 week vs. 12^th^ week	0 week vs. 24^th^ week
Serum zinc (µg/dl)	79.4±46.3	87.3±38.3	99.3±27.8	0.380	0.154
Serum copper (µg/dl)	176.3±41.2	136.9±50.9	104.9±26.0	0.003	0.001

Table 4 shows the mean serum zinc and copper levels in different disease activity states among JIA patients. Serum zinc levels showed no significant differences among patients with disease activity levels at both the 12th and 24th weeks. However, serum copper levels showed a significant difference among disease activity levels at both the 12th (p < 0.05) and 24th weeks (p < 0.05) of follow-up.

**Table 4:** Mean serum zinc and copper in different disease activity levels among juvenile idiopathic arthritis patients.

	Disease activity				P-value
	High, mean±SD	Moderate, mean±SD	Low, mean±SD	Inactive, mean±SD	
12 weeks	n=14	n=11	n=13	n=7	
Serum zinc (µg/dl)	77.1±28.0	80.1±38.8	94.1±44.6	106.3±41.0	0.327
Serum copper (µg/dl)	162.3±43.4	157.8±60.2	112.1±28.7	99.5±44.7	0.001
24 weeks	n=3	n=7	n=12	n=23	
Serum zinc (µg/dl)	73.7±3.2	97.6±16.8	91.5±35.0	103.5±27.9	0.303
Serum copper (µg/dl)	157.1±12.9	108.6±18.9	108.2±28.8	100.7±25.3	0.008

## Discussion

JIA is the most common chronic rheumatic disease in childhood, which is a leading cause of short and long-term disability [14]. Zinc and copper play a crucial role in chronic inflammatory arthritis because they are involved in key metabolic processes within joint tissues and influence immune system activity [5,6]. The study aimed to assess the serum zinc and copper levels and to see their association with the different disease activity states in JIA patients. Hence, fluctuations in these minerals could serve as valuable biomarkers indicating the disease activity states of JIA patients.

The mean age of JIA patients was 9.8 ± 3.32 years in this study, which corresponds to another Bangladeshi study, where the mean age was 9.3 years, and a Swedish study, where the mean age was found to be nine years, which is also consistent with the present study [15,16]. These study findings explained the juvenile onset of the disease in most of the contexts.

A Bangladeshi study showed that the male-to-female ratio was 1.3:1, where 58% were male and 42% were female [17], which was relevant to this study, where samples were found to be slightly male predominant, with a male-to-female ratio of 1.14:1. Male predominance (58.2%) was also observed in an Indian study [18]. Increased numbers of female patients can be due to a higher chance of autoimmune disease in girls.

Studies in this subcontinent show a male predominance, possibly due to our socio-cultural background. The emphasis placed by parents on male children may lead to their earlier and more frequent hospital visits compared to females.

In the present study, different subtypes of JIA patients were reported. Among them, ERA was the most common (38%) subtype, followed by sJIA (31%), oligoarticular JIA, polyarticular RF-negative JIA, and polyarticular RF-positive JIA, which corresponds to other studies in this subcontinent where ERA was the most common subtype [18-20]. The high frequency of ERA in these studies may be attributed to several factors, including a predominance of males, parents’ tendency to seek early medical attention for boys, and a higher prevalence of this subtype in the Asian subcontinent. In contrast, studies conducted in Europe, North America, and Africa indicate that oligoarthritis is the largest subtype of JIA [21,22].

The current study showed that all patients had high disease activity (100%) at the initial visit, and among them, 15.6% and 51.1% of JIA patients showed inactive disease states at 12th week and 24th week of follow-up, respectively. This corresponds to another Bangladeshi study, where most of the patients (98%) had severe disease activity at the initial visit due to severe manifestations at presentation, and at follow-up after six months of treatment, 40% of them were maintaining inactive disease status [17].

In this study, at the initial visit, serum Zn level was lower than normal, and serum Cu level was higher than normal. The comparison of mean serum zinc and copper was done at the 12th and 24th weeks of follow-up among the study population. There was an increase in serum zinc levels in the subsequent 12th and 24th weeks of follow-up, compared to the initial visit. However, these changes were not statistically significant.

On the contrary, there was a notable decline in serum copper levels observed at subsequent follow-up, and changes were statistically significant for 0 weeks vs. 12 weeks, and for 0 weeks vs. 24 weeks. A follow-up study on serum zinc and serum copper estimation in JIA patients has not yet been found in the literature to compare with the present study.

In a cross-sectional study conducted in Bangladesh, serum zinc and serum copper levels were measured in JIA patients and compared with a control group. The study reported that mean serum zinc levels were reduced (84.6 ± 39.8) in comparison with controls (101.4 ± 25.3), but it was not statistically significant. On the contrary, serum copper levels were raised in JIA patients (121.9 ± 42.83) than in controls (88.6 ± 8.9) in the active disease cohort, which was statistically significant [23]. This result corresponds with other studies conducted in Egypt and Brazil [7,11].

In this study, the mean serum zinc and copper levels were also assessed and compared in different disease activity states among JIA patients. Serum zinc levels gradually increased to normal with different disease activity levels in the 12th week and 24th week, but there were no statistically significant differences observed. However, serum copper levels showed a significant difference among disease activity levels at the 12th week and 24th week. Specifically, patients with high disease activity had higher mean serum copper levels compared to patients with moderate, low, and inactive disease. No studies have been found in the literature that assess serum zinc and copper levels in association with disease activity in JIA patients, which is comparable to the present study.

Strengths and limitations

This study supports that the use of serum copper level is a useful biomarker for the disease activity of JIA patients as measured by JADAS-27. The prospective observational design facilitated simultaneous data collection and monitoring, which reduced recall error. The absence of a healthy control group for baseline comparison is an important limitation. Non-significant zinc level needs further investigation on a larger sample size and across different JIA subtypes. The heterogeneity of treatment regimens during follow-up with nonsteroidal anti-inflammatory drugs, steroids, or methotrexate, as well as potential dietary and nutritional influences, may alter zinc and copper levels; however, these factors were not addressed in the present study.

## Conclusions

Serum Zn and Cu levels were altered in different disease activity states of JIA patients. Specifically, serum Cu was notably elevated during active disease states, while serum Zn showed a decrease, though this change was not statistically significant. The study supports the potential utility of serum copper levels as a biomarker for monitoring disease activity and treatment response in JIA patients, as measured by JADAS.
